# The association of biological age and its trajectory with incident heart failure: a cohort study from China

**DOI:** 10.3389/fcvm.2025.1651743

**Published:** 2026-01-22

**Authors:** Yuhao Hu, Huayu Sun, Chenrui Zhu, Jing Hu, Jintao Tao, Bo Li, Qianxun Cai, Yutong Wu, Shuohua Chen, Shouling wu, Yuntao Wu

**Affiliations:** 1 Hebei North University, Zhangjiakou, Hebei, China; 2 Kailuan General Hospital, Tangshan, China; 3 Public Health Department, Ngari Prefecture People’s Hospital, Ngari Prefecture, Xizang, China; 4 School of Public Health, North China University of Science and Technology, Tangshan, China; 5 Department of Cardiology, Kailuan Hospital, North China University of Science and Technology, Tangshan, China; 6 University of Toronto, Toronto, ON, Canada

**Keywords:** aging trajectory, biological age, cohort study, heart failure, kailuan study

## Abstract

**Background:**

Research on biological age focused on the optimization and upgrading of aging clocks, which can now prospectively predict a variety of diseases. The biological age (BA) based on clinical parameters has shown predictive value for cardiovascular disease. However, evidence linking BA and its trajectories with heart failure (HF) remained limited. This study aimed to construct a clinical-parameter-based BA and to investigate its association, along with BA trajectories, with incident heart failure.

**Methods:**

This study utilized data from the Kailuan Study, which included 76,908 Chinese adults who underwent their first health examination between 2006 and 2007. A deep neural network model was employed to estimate BA based on 32 clinical indicators. Participants were stratified into three groups-decelerated aging, accelerated aging, and normal aging-according to their baseline BA values. Six distinct aging trajectories were subsequently identified using data from the first three follow-up examinations. Cox proportional hazard models were applied to estimate hazard ratios (HRs) and 95% confidence intervals (CIs) for the associations between aging status or BA trajectories and HF incidence.

**Results:**

Participants exhibiting accelerated aging demonstrated a 30% higher risk of HF (HR: 1.30; 95%CI: 1.19–1.43) compared to those with normal aging. Conversely, those following a high-stable trajectory demonstrated the highest risk of HF (HR: 1.79; 95%CI: 1.48–2.17). Additionally, when compared to the high-stable trajectory, the high-descending trajectory was linked to a significantly lower risk of HF (HR: 0.74; 95%CI: 0.60–0.91).

**Conclusions:**

Accelerated biological aging significantly increased the risk of HF, whereas decelerated biological aging was linked to a reduced risk of HF. Individuals who consistently exhibited a higher level of biological aging were at the greatest risk for HF.

## Introduction

Aging constituted a significant risk factor for age-related chronic diseases, cognitive decline, and mortality. Chronological age (CA), serving as an indicator of individual aging status, was the primary contributor to the Framingham Risk Score (1) and represented the most robust predictor of cardiovascular disease. Nevertheless, owing to variations in epigenetics (2), nutritional intake (3), environmental exposure (4), and access to healthcare (5, 6), individuals with identical CA may have exhibited disparate rates of biological aging. The concept of biological age (BA) was introduced in 1988 (7) to delineate the true pace of aging. Precisely quantifying BA could facilitate the identification of early physiological alterations and elucidated the heterogeneity inherent in the aging process across different individuals.

Recent research had increasingly emphasized the estimation of BA based on clinical and biochemical parameters. Cohort studies had demonstrated that BA was more precise than CA in predicting age-related events. In comparison with omics-based measures of BA, such as DNA methylation age (8, 9), telomere length (10), and the inflammatory aging clock (11), BA derived from clinical indicators was more cost-effective, accessible, and scalable. Furthermore, compared to phenotype-based measures of BA, such as the Healthy Aging Index (12) and Frailty Index (13, 14), clinical-parameter-based BA could better reflect early physiological changes, captured the complexity of the aging process, and exhibited good applicability across both young and elderly populations (15). Common methods for estimating BA include standard linear models, the Klemera and Doubal method (KDM), homeostatic dysregulation (HD), and deep neural networks (DNNs) (16). Among these, DNNs were especially suited for training large datasets comprising tens of thousands or more samples. By explicitly encoding specific biological pathways into neural network architectures or backpropagating information to input features (17), DNNs may have more effectively captured the intricacies of the aging process.

Cohort studies had demonstrated that BA was a reliable predictor of cardiovascular disease, cancer, and all-cause mortality (18–20), but the majority of evidence originated from Western populations, with limited focus on Asian populations. Research exploring the association between BA and the risk of heart failure (HF) remained scarce. Additionally, investigations into heterogeneous aging trajectories had predominantly centred on the relationships between frailty trajectories and mortality in elderly populations (21). To date, no study had examined the relationship between BA trajectories and HF risk across a broad age spectrum. To address this research gap, the present study utilized data from the Kailuan Study, focusing on Chinese adults aged 18 years or older. BA was estimated using a DNNs model. It aimed to address the following core scientific questions: First, it employed a deep neural network (DNN) model to calculate the biological age of the target population, systematically explored the longitudinal association between biological age and the incidence risk of heart failure (HF), and filled the existing research gap regarding the association between these two factors in Asian populations. Second, taking into account the dynamic changes in biological age over time, it further constructed individual-specific biological age trajectories, clarified the association patterns between different aging trajectory types and the subsequent incidence risk of HF, and provided a novel theoretical basis for HF risk stratification and early intervention in the general population across all age groups.

## Methods

### Study population

The Kailuan study was a prospective cohort study conducted in the Kailuan community in Tangshan, Republic of China (Trial Registration Number: ChiCTR-TNRC-11001489), which is a large, modern city southeast of Beijing. Detailed study design and procedures had been described in detail (22, 23). Since June 2006, a total of 101,510 adult participants, including 81,110 men and 20,400 women, were enrolled from 11 hospitals in the Kailuan community and underwent questionnaire assessments, clinical examinations, and laboratory tests. Follow-up assessments have been conducted biennially, while the database for cardiovascular disease incidence, tumor incidence, and all-cause mortality has been updated annually. By the end of 2022, the Kailuan Community had completed eight rounds of follow-up, involving approximately 800,000 person visits.

In this study, the baseline BA data were obtained from the survey conducted between 2006 and 2007. BA trajectories were subsequently constructed using data collected during the periods of 2006–2007, 2008–2009, and 2010–2011. The final follow-up was conducted on December 31, 2022.

**Figure 1:**
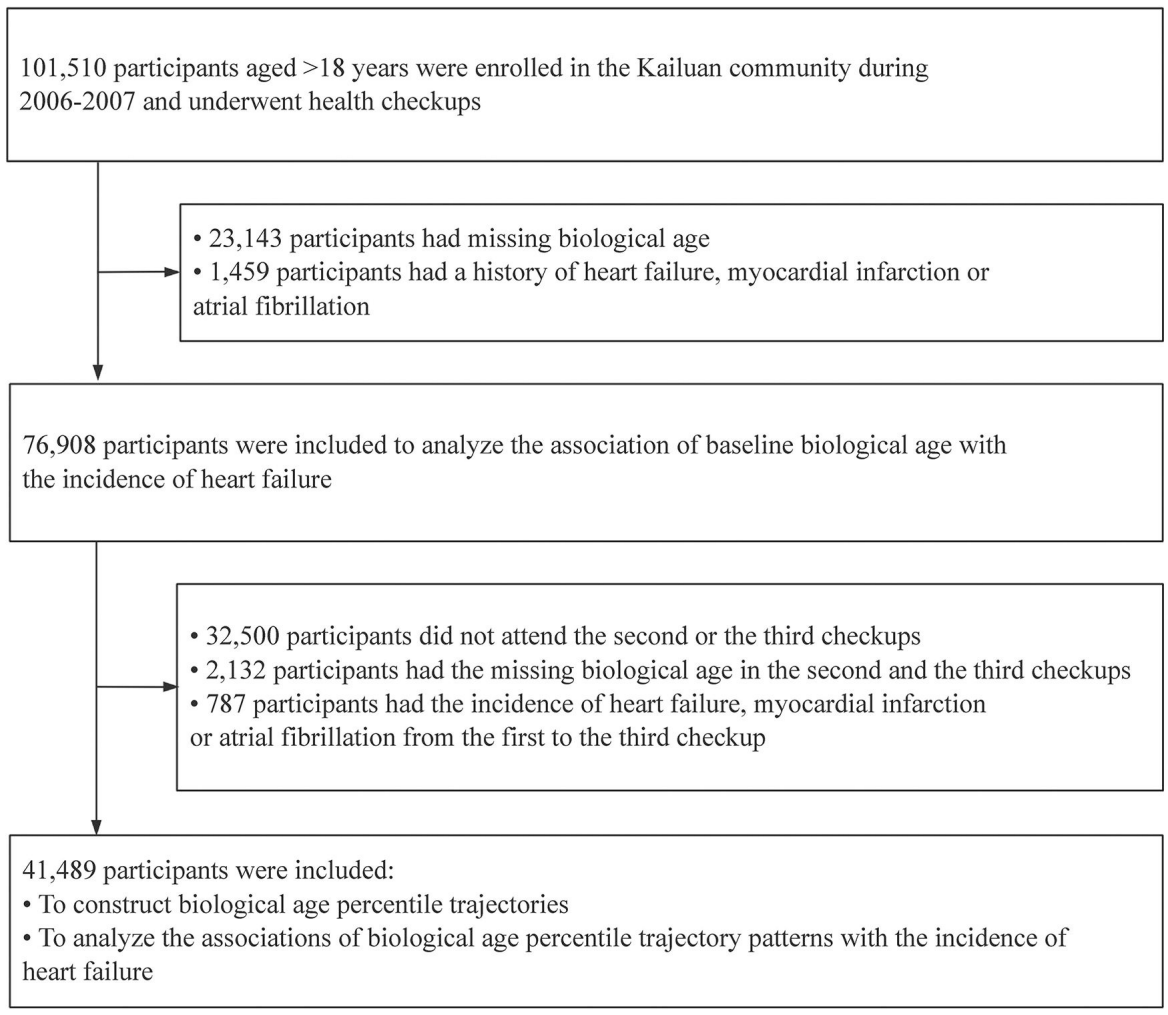
Flow chart of subject selection process.

This study initially included 76,908 participants who had no history of HF, myocardial infarction, or atrial fibrillation and had complete baseline data between 2006 and 2007 to investigate the association between biological aging status and risk of HF. Subsequently, we further excluded participants who did not participate in either the 2008–2009 or 2010–2011 survey, as well as those lacking BA data during the 2008–2009 and 2010–2011 survey. Additionally, participants diagnosed with new-onset HF, myocardial infarction, or atrial fibrillation between 2006 and 2010 were also excluded. Ultimately, a cohort including 41,489 participants was used to construct BA trajectories and analyze the association between BA trajectory patterns and the risk of HF. The detailed flowchart for participant inclusion and exclusion is presented in Figure 1.

This study was conducted in accordance with the Helsinki Declaration of the World Medical Association and the Reporting Guidelines for Observational Epidemiological Studies. Ethical approval was obtained from the Ethics Committee of Kailuan General Hospital (Protocol Number: 2021012). All participants agreed to participate in the Kailuan study and signed an informed consent form.

### Assessment of outcomes

The primary outcome of this study was new-onset HF. Consistent with prior studies (24, 25), the International Classification of Diseases and Related Health Problems (10th Revision) (ICD-10) was utilized to identify cases of HF (I50.x). Trained medical personnel collected data on HF incidence through discharge summaries, death certificates, or medical insurance records from 11 hospitals. A team of cardiovascular specialists reviewed and confirmed suspected cases of HF. The definition of HF referred to the diagnostic criteria outlined in the “2018 Chinese Guidelines for Diagnosis and Treatment of Heart Failure” (26). Confirmation of HF required medical records to satisfy the following criteria: (1) Presence of HF symptoms, such as dyspnea, fatigue, and fluid retention, with discharge records indicating New York Heart Association (NYHA) functional class II reviewed and confirmed sus (2) A left ventricular ejection fraction (LVEF) of ≤50% measured by two-dimensional and Doppler echocardiography using the modified Simpson method; (3) Elevated levels of plasma N-terminal pro-brain natriuretic peptide (NT-proBNP). A diagnosis of HF required fulfilment of criterion (1) along with either criterion (2) or (3).

### Biological Age

We extracted 32 indicators reflecting the function, structure, and/or overall health status of the cardiovascular, liver, kidney, immune, and metabolic systems from the Kailuan Research Database. A DNNs model was employed to develop and train the BA estimation algorithm. Detailed procedures and results of the model construction had been reported in prior literature (27). Specifically, BA models were independently constructed for the surveys conducted during the periods of 2006–2007, 2008–2009, and 2010–2011.

We used normalization methods to construct BA percentiles to eliminate the influence of CA. Participants were stratified by CA in one-year intervals, with those under 26 or over 76 grouped together due to small sample sizes. Within each CA stratum, BA was ranked to determine the corresponding percentile value. Given the significant sex-related differences in BA distribution, participants in each stratum were classified into three aging status groups based on sex-specific BA percentiles: Decelerated aging: BA < Q1; Normal aging: Q1 ≤ BA ≤ Q3; Accelerated aging: BA > Q3.

### Aging trajectory assessment

**Figure 2:**
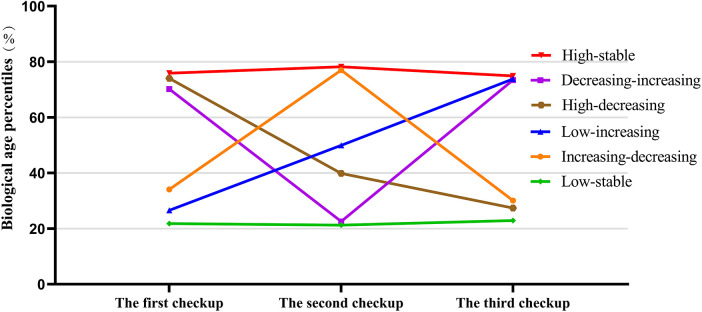
Mean biological Age percentiles in the first, second, and third checkup, according to 6 biological Age percentile trajectory patterns. Biological age percentile, biological age was ranked from lowest to highest in chronological age strata, and the corresponding cumulative percentile was calculated.

We employed group-based trajectory modelling using the SAS Proc Traj procedure (39–41), specifying a censored normal distribution to identify subgroups with similar latent trajectories of biological age percentiles between 2006 and 2010. Models with 6, 5, 4, 3, and 2 trajectory groups were fitted sequentially and compared using the Bayesian Information Criterion (BIC). The six-trajectory model demonstrated the best overall fit. Subsequently, we tested various functional forms—linear and quadratic—to determine the optimal trajectory shapes. Finally, based on the smallest absolute BIC value, average posterior probability greater than 0.7, and a minimum group membership proportion exceeding 5%, the final model was selected. It consisted of six distinct trajectories, including two linear and four quadratic functions (Figure 2).

### Assessment of potential covariates

All participants underwent standardized assessments performed by trained medical personnel at 11 hospitals within the Kailuan community. Evaluations were conducted using uniform protocols, instruments, and reagents. Demographic characteristics (CA, sex, education level, and occupation), lifestyle factors (physical activity, smoking, alcohol consumption, salt intake), medical history, and family history were collected using a structured questionnaire. Education level was categorized as high school or below vs. college or above. The occupation was classified as a coal miner or other. Smoking and drinking status were categorized as never, former, or current. Physical activity levels were defined as low (<10 min/week), moderate (10–80 min/week), or high (>80 min/week). Salt intake was categorized into low, medium, and high. Income level was divided into <1,000 CNY/month and ≥1,000 CNY/month.

Anthropometric and physiological measurements—including height, weight, waist circumference, hip circumference, and systolic and diastolic blood pressure—were taken by trained staff following standard procedures. Fasting venous blood samples (>8 h fasting) were collected and analyzed for biochemical and hematological parameters using automated analyzers (Hitachi 747 and Sysmex XT-1800i). Detailed laboratory procedures are available in Supplementary Table S1.

### Statistical analysis

Continuous variables with a normal distribution were presented as means ± standard deviation and compared using analysis of variance. Skewed continuous variables were expressed as medians with interquartile ranges (P25–P75) and compared using the Kruskal–Wallis test. Categorical variables were presented as frequencies and percentages and compared using the Chi-square test. Missing covariate data were imputed using multivariate chained equations.

Person-years of follow-up for HF were calculated from the 2006–2007 survey (for baseline analysis) or from the 2010–2011 survey (for trajectory analysis) to the date of incident HF, death, or the last follow-up (December 31, 2022), whichever came first. Cox proportional hazard models were used to estimate hazard ratios (HRs) and 95% confidence intervals (CIs) for the association between baseline biological aging status or BA percentile trajectories and the risk of HF. Proportional hazard assumptions were assessed using Schoenfeld residual plots.

Adjusted covariates included the following data from the 2006–2007 or 2010–2011 survey: CA, sex, education level (high school or below vs. college or above), occupation (coal miner or other), physical activity level (low, moderate, or high), smoking status (never, former, or current), alcohol drinking status (never, former, or current), salt intake (low, medium, or high), and income level (<1,000 CNY/month or ≥1,000 CNY/month).

Subgroup analyses were conducted stratified by age (<45, 45–65, or ≥65 years), sex, education level, occupation, physical activity, smoking status, alcohol consumption, salt intake, and income level to investigate potential effect modification in the association between baseline aging status and HF.

To address potential reverse causality, participants who developed HF within the first year of follow-up were excluded. Subsequently, the associations between baseline aging status and BA percentile trajectories with HF risk were reanalyzed. Additionally, non-HF-related mortality was treated as a competing event, and Fine-Gray competing risk models were constructed accordingly.

All statistical analyses were performed using SAS version 9.4. All tests were two-sided, and a *p*-value < 0.05 was considered statistically significant.

## Results

The baseline characteristics of the 76,908 participants according to different aging status were summarized in Supplementary Table S2. The average CA was 51.3 years, and 61,115 participants (79.5%) were male. Compared with the other groups, participants with accelerated aging had the highest proportion of lower education levels and high salt intake.

The demographic and clinical characteristics of 41,489 participants included in the construction of BA percentile trajectories were presented in Table 1. In the 2010–2011 survey, the mean CA was 52.9 years, with 31,798 (76.6%) being male. Compared to individuals in the low-stable trajectory group, participants in the high-stable trajectory group were more likely to have lower educational attainment, current smoking and drinking habits, and lower income levels.

**Table 1:** Basic characteristics of 41,489 participants according to the biological Age percentile trajectory patterns^a^.

Characteristics	Low-stable	Increasing-decreasing	Low-increasing	High-decreasing	Decreasing-increasing	High-stable	*P* value
No. of participants (%)	10,130 (24.42)	4,552 (10.97)	6,123 (14.76)	6,016 (14.50)	3,109 (7.49)	11,559 (27.86)	
Chronological age, mean (SD), y	52.9 ± 11.8	52.8 ± 11.8	52.9 ± 11.7	52.8 ± 11.8	52.6 ± 12.0	53.0 ± 11.8	0.6995
Biological age, mean (SD), y	46.4 ± 7.5	48.1 ± 7.2	56.3 ± 7.1	47.6 ± 7.0	55.9 ± 7.3	56.8 ± 7.5	<.0001
Gender, No. (%)							<.0001
Female	2,421 (23.9)	1,088 (23.9)	1,361 (22.2)	1,277 (21.2)	782 (25.2)	2,762 (23.9)	
Male	7,709 (76.1)	3,464 (76.1)	4,762 (77.8)	4,739 (78.8)	2,327 (74.8)	8,797 (76.1)	
Educational level, No. (%)							<.0001
High school or below	8,865 (87.5)	4,138 (90.9)	5,390 (88.0)	5,485 (91.2)	2,850 (91.7)	10,565 (91.4)	
College or above	1,265 (12.5)	414 (9.1)	733 (12.0)	531 (8.8)	259 (8.3)	994 (8.6)	
Occupation, no. (%)							<.0001
Coal miner	3,244 (32.0)	1,371 (30.1)	1,726 (28.2)	2,190 (36.4)	974 (31.3)	3,074 (26.6)	
Other	6,886 (68.0)	3,181 (69.9)	4,397 (71.8)	3,826 (63.6)	2,135 (68.7)	8,485 (73.4)	
Physical activity, no. (%)							<.0001
Low intensity	3,588 (35.4)	1,423 (31.3)	1,970 (32.2)	2,156 (35.8)	1,039 (33.4)	3,648 (31.6)	
Moderate intensity	5,092 (50.3)	2,475 (54.4)	3,326 (54.3)	2,941 (48.9)	1,656 (53.3)	6,179 (53.5)	
High intensity	1,450 (14.3)	654 (14.4)	827 (13.5)	919 (15.3)	414 (13.3)	1,732 (15.0)	
Smoking status, no. (%)							0.0006
Never	6,297 (62.2)	2,828 (62.1)	3,790 (61.9)	3,532 (58.7)	1,925 (61.9)	7,078 (61.2)	
Quit	456 (4.5)	175 (3.8)	261 (4.3)	279 (4.6)	123 (4.0)	549 (4.7)	
Currently	3,377 (33.3)	1,549 (34.0)	2,072 (33.8)	2,205 (36.7)	1,061 (34.1)	3,932 (34.0)	
Alcohol consumption status, no. (%)							<.0001
Never	6,665 (65.8)	2,984 (65.6)	4,004 (65.4)	3,740 (62.2)	2,006 (64.5)	7,460 (64.5)	
Quit	49 (0.5)	8 (0.2)	24 (0.4)	44 (0.7)	17 (0.5)	77 (0.7)	
Currently	3,416 (33.7)	1,560 (34.3)	2,095 (34.2)	2,232 (37.1)	1,086 (34.9)	4,022 (34.8)	
Salt intake, no. (%)							<.0001
Low and salt intake	1,956 (19.3)	777 (17.1)	923 (15.1)	1,101 (18.3)	521 (16.8)	1,966 (17.0)	
Medium and salt intake	7,066 (69.8)	3,225 (70.8)	4,592 (75.0)	4,252 (70.7)	2,296 (73.9)	8,461 (73.2)	
High and salt intake	1,108 (10.9)	550 (12.1)	608 (9.9)	663 (11.0)	292 (9.4)	1,132 (9.8)	
Income level, no. (%)							0.1032
<$132 /month	4,735 (46.7)	2,224 (48.9)	2,967 (48.5)	2,817 (46.8)	1,486 (47.8)	5,504 (47.6)	
≥$132 /month	5,395 (53.3)	2,328 (51.1)	3,156 (51.5)	3,199 (53.2)	1,623 (52.2)	6,055 (52.4)	

a Biological age percentile, biological age was ranked from lowest to highest in chronological age strata, and the corresponding cumulative percentile was calculated.

During follow-up from 2006 to 2022 (median follow-up duration: 16 years; interquartile range: 15.61–16.18 years), a total of 2,338 participants developed HF. The incidence of HF was highest among participants exhibiting accelerated aging (2.64 per 1,000 person-years) and lowest among those with decelerated aging (1.54 per 1,000 person-years). Compared to participants with normal aging, those with accelerated aging demonstrated an adjusted HR of 1.30 (95%CI: 1.19–1.43), while those with decelerated aging exhibited an adjusted HR of 0.76 (95% CI: 0.68–0.84) (Table 2).

**Table 2:** Association of baseline aging Status with the risk of heart failure^a^.

Characteristics	Baseline aging status, HR (95% CI)		
	Aging deceleration	Aging normal	Aging acceleration
Heart failure			
Cases, no. (%)	443 (2.31)	1,154 (3.00)	740 (3.85)
Absolute incident rate, per 1,000 person-years	1.54	2.02	2.63
Model 1^b^	0.76 (0.68-0.85)	Ref	1.30 (1.19–1.43)
PAF (95% CI)	−0.56 (−0.75 to 0.35)	Ref	1.14 (0.73–1.63)
Model 2^c^	0.76 (0.68-0.84)	Ref	1.30 (1.19–1.43)
PAF (95% CI)	−0.56 (−0.75 to 0.37)	Ref	1.14 (0.73–1.63)
Fine-Gray^d^			
Model 1^a^	0.77 (0.69–0.86)	Ref	1.28 (1.17–1.41)
Model 2^b^	0.77 (0.69–0.86)	Ref	1.28 (1.17–1.40)

CI, confidence interval; HR, hazard ratio; PAF, population attributable fraction.

a Aging deceleration, less than the first biological age quartile; aging normal, ranged from the second to third biological age quartile; aging acceleration, higher than the third biological age quantile.

b Model 1 adjusted for chronological age and gender.

c Model 2 included covariates in model 1 and education level (high school or below or college or above), occupation (coal miner or other), physical activity (low intensity, moderate intensity, or high intensity), smoking status (never, quit, or currently), alcohol consumption (never, quit, or currently), salt intake (low salt intake, moderate salt intake, or high salt intake), and income level [income <1,000 Chinese Yuan ($132) /month or income ≥1,000 Chinese Yuan/month].

d Fine-Gray: Non-HF-related mortality was treated as a competing event, and Fine-Gray competing risk models were constructed accordingly.

The association between baseline aging status and HF risk was more pronounced among younger participants (<65 years) compared to those aged 65 years or older (P for interaction <0.01). Additionally, no significant interactions were observed between baseline aging status and other covariates (all *P* > 0.05) (Supplementary Figure S1).

Compared with the traditional risk model, models that incorporate BA (C-statistic: 0.7490; 95% CI: 0.7399–0.7581) or baseline aging status (C-statistic: 0.7429; 95% CI: 0.7336–0.7521) demonstrated enhanced predictive performance. However, an enhancement in discrimination ability was observed only in the model that incorporated BA (NRI: 0.1869; 95% CI: 0.1460–0.2278) (Supplementary Table S3).

During follow-up from 2010 to 2022 (median follow-up duration: 12.03 years, interquartile range: 11.66–12.32 years), a total of 858 participants developed HF. The HF incidence was highest in the high-stable trajectory group (2.41 per 1,000 person-years) and lowest in the low-stable group (1.34 per 1,000 person-years). Compared with the low-stable group, all other trajectory groups except for the increasing-decreasing group had significantly higher risks of HF. The highest risk was observed in the high-stable group (HR: 1.79, 95% CI: 1.48–2.17), followed by the decreasing-increasing group (HR: 1.45, 95% CI: 1.09–1.93), the high-decreasing group (HR: 1.32, 95% CI: 1.04–1.67), and the low-increasing group (HR: 1.30, 95% CI: 1.03–1.65) (Table 3). Compared with the high-stable group, the high-decreasing trajectory was associated with a lower risk of HF (HR: 0.74, 95% CI: 0.60–0.91), whereas the decreasing-increasing trajectory did not show a significant difference. No significant differences in HF risk were observed between the other trajectory comparisons (decreasing-increasing vs. high-decreasing; increasing-decreasing vs. low-increasing) (Table 4).

**Table 3:** Association of biological Age percentile trajectory patterns with the risk of heart failure^a^.

Characteristics	Aging trajectory patterns, HR (95% CI)					
	Low-stable	Increasing-decreasing	Low-increasing	High-decreasing	Decreasing-increasing	High-stable
Heart failure						
Cases, no. (%)	157 (1.55)	78 (1.71)	121 (1.98)	121 (2.01)	67 (2.16)	314 (2.72)
Absolute incident rate, per 1,000 person-years	1.34	1.49	1.73	1.75	1.89	2.41
Model 1^b^	Ref	1.13 (0.86–1.48)	1.30 (1.03–1.65)	1.33 (1.05–1.68)	1.45 (1.09–1.94)	1.80 (1.48–2.18)
PAF (95% CI)	Ref	0.20 (−0.22–0.74)	0.59 (0.06–1.27)	0.66 (0.10–1.35)	0.96 (0.19–1.99)	2.13 (1.29–3.11)
Model 2^c^	Ref	1.12 (0.86–1.47)	1.30 (1.03–1.65)	1.32 (1.04–1.67)	1.45 (1.09–1.93)	1.79 (1.48–2.17)
PAF (95% CI)	Ref	0.19 (−0.22–0.72)	0.59 (0.06–1.27)	0.64 (0.08–1.33)	0.96 (0.19–1.97)	2.10 (1.29–3.09)
Fine-Gray^d^						
Model 1^b^	Ref	1.11 (0.85–1.46)	1.28 (1.01–1.62)	1.30 (1.03–1.65)	1.41 (1.06–1.88)	1.75 (1.44–2.12)
Model 2^c^	Ref	1.11 (0.85–1.46)	1.28 (1.01–1.63)	1.29 (1.02–1.64)	1.40 (1.05–1.87)	1.74 (1.44–2.11)

CI, confidence interval; HR, hazard ratio; PAF, population attributable fraction.

a Biological age percentile, biological age was ranked from lowest to highest in chronological age strata, and the corresponding cumulative percentile was calculated.

b Model 1 adjusted for chronological age and gender.

c Model 2 included covariates in model 1 and education level (high school or below or college or above), occupation (coal miner or other), physical activity (low intensity, moderate intensity, or high intensity), smoking status (never, quit, or currently), alcohol consumption (never, quit, or currently), salt intake (low salt intake, moderate salt intake, or high salt intake), and income level [income <1,000 Chinese Yuan ($132) /month or income ≥1,000 Chinese Yuan/month].

d Fine-Gray: Non-HF-related mortality was treated as a competing event, and Fine-Gray competing risk models were constructed accordingly.

**Table 4:** Group comparisons incidence of heart failure according to biological age percentile trajectory patterns^a^.

Characteristics	Cases, no. (%)	Incident rate, per 1,000 person-years	Model 1^b^ HR (95%CI)	Model 2^c^ HR (95%CI)
Heart failure				
High-stable vs. high-decreasing				
High-stable	314 (2.72)	2.41	Ref	Ref
High-decreasing	121 (2.01)	1.75	0.74 (0.60–0.91)	0.74 (0.60–0.91)
High-stable vs. decreasing-increasing				
High-stable	67 (2.16)	2.41	Ref	Ref
Decreasing-increasing	314 (2.72)	1.89	0.81 (0.62–1.05)	0.81 (0.62–1.05)
High-decreasing vs. decreasing-increasing				
Decreasing-increasing	67 (2.16)	1.89	Ref	Ref
High-decreasing	121 (2.01)	1.75	0.91 (0.68–1.23)	0.91 (0.68–1.23)
Low-increasing vs. increasing-decreasing				
Increasing-decreasing	78 (1.71)	1.49	Ref	Ref
Low-increasing	121 (1.98)	1.73	1.16 (0.87–1.54)	1.16 (0.87–1.54)

CI, confidence interval; HR, hazard ratio.

a Biological age percentile, biological age was ranked from lowest to highest in chronological age strata, and the corresponding cumulative percentile was calculated.

b Model 1 adjusted for chronological age and gender.

c Model 2 included covariates in model 1 and education level (high school or below or college or above), occupation (coal miner or other), physical activity (low intensity, moderate intensity, or high intensity), smoking status (never, quit, or currently), alcohol consumption (never, quit, or currently), salt intake (low salt intake, moderate salt intake, or high salt intake), and income level [income <1,000 Chinese Yuan ($132) /month or income ≥1,000 Chinese Yuan/month].

Sensitivity analyses were conducted by excluding participants who developed HF within the first year of follow-up and by applying competing risk models to account for the impact of all-cause mortality. The associations between baseline aging status, BA percentile trajectories, and HF risk remained consistent with the primary findings (Supplementary Tables S4–S7).

## Discussion

Our research findings revealed a substantial association between baseline aging status, as defined by BA, and the risk of developing HF. Specifically, individuals exhibiting accelerated aging demonstrated a higher risk of developing HF compared to those with normal aging patterns. Conversely, individuals with decelerated aging exhibited a reduced risk of HF. Furthermore, when compared to participants who consistently maintained the lowest percentile of BA (low-stable trajectory), other trajectory patterns-excluding the increasing-decreasing trajectory-were associated with an elevated risk of HF. Notably, the highest risk of HF was observed within 4 years among individuals maintaining the highest percentile of BA (high-stable trajectory). Additionally, participants whose trajectories transitioned from high to lower BA percentiles (high-decreasing trajectory) exhibited a diminished risk of HF relative to those in the high-stable group.

To our knowledge, this was the first large-scale prospective cohort study to demonstrate a longitudinal association between BA and HF risk. Previous studies had used different indicators and methods to construct phenotype age and other BAs and validated their predictive ability for age-related events in multiple cohorts based on Eastern and Western populations (19, 28–30). Mamoshina et al. trained independent DNNs to construct hematological aging clocks for South Korean, Canadian, and Eastern European populations, thereby predicting the risk of all-cause mortality across different populations (31). A study based on Moli Sani further extended this evidence to other age-related events, demonstrating that BA calculated using 36 circulating biomarkers and trained through DNNs could predict the risk of specific causes of mortality, hospitalization, cardiovascular disease, and cancer (32). However, the application of DNNs-derived BA in predicting HF has not been previously documented in the literature. This study provides novel evidence from a Chinese adult population, revealing heterogeneity in BA among individuals with the same CA and establishing that accelerated aging served as a risk factor, whereas decelerated aging acted as a protective factor against HF.

The association between aging status and the risk of developing HF remained stable across both male and female populations, which was consistent with prior studies on BA and age-related events (33). However, our findings indicated that this association was more pronounced in younger, predominantly middle-aged populations. A cohort study revealed that BA was strongly associated with all-cause mortality across multiple age groups, including young, middle-aged, and elderly individuals (15). Two additional studies demonstrated that the correlation between the frailty index and the risk of all-cause mortality was stronger in younger populations compared to older ones (13, 34). These results aligned with those obtained using continuous BA to estimate HF risk across different age groups. While incorporating CA into the calculation of BA could better reflect an individual's physiological state, it may have introduced bias in risk analysis, even when CA was adjusted for in the model. In our study, normalization methods were employed to eliminate the potential influence of CA, revealing a significant correlation between aging status and the risk of developing HF in the middle-aged population (45–65 years old). Accelerated aging represented a risk factor across all age groups.

Our research further demonstrated that BA could enhance the predictive power of traditional models, which included CA, gender, education level, occupation, physical activity, smoking status, alcohol consumption, salt intake, and income level. Two cohort studies conducted in Asian populations had shown that BA, derived from blood biomarkers, significantly improves mortality prediction (30, 35). Liu et al. calculated BA among adults aged 20 and above in the Third National Health and Nutrition Examination Survey (NHANES IV) and revealed that BA provided additional value for predicting 10-year mortality across all age groups (15). By integrating indicators reflecting the structure and function of the cardiovascular, hepatic, renal, immune, and metabolic systems into the assessment model for health risk, we achieved a more comprehensive understanding of physiological status, thereby enhancing health management and enabling precision prevention.

Our study identified six heterogeneous trajectories of BA percentile patterns and investigated their association with HF. Prior research on the relationship between BA and aging-related events had predominantly focused on single-point BA measurements, with limited studies examining the effects of continuous BA changes over time. The Health ABC study quantified changes in the Healthy Aging Index over a 9-year period and identified four distinct change patterns. Among these, the pattern with the most pronounced increase was associated with the highest mortality risk (36). A large-scale prospective MJ cohort study calculated multidimensional aging measures (MDAges) and delineated three homogeneous accelerated aging trajectories. The finding indicated that moderate or high accelerated aging trajectories were significantly associated with an elevated risk of mortality compared to low accelerated aging trajectories (37). A cohort study focusing on elderly individuals constructed five heterogeneous frailty trajectories based on frailty traits. Specifically, compared with the moderate degrading trajectory, the moderate increasing trajectory, high stable trajectory, and high increasing trajectory were associated with a higher risk of mortality (21). Additionally, another study conducted among older adults identified multiple sets of frailty trajectories across different age groups. This study also demonstrated that the increase in frailty was significantly associated with an elevated risk of all-cause mortality (38). Our study revealed that the risk of HF was lowest in individuals following low-stable aging trajectories, underscoring the critical importance of maintaining consistently low and stable aging status for HF prevention. The risk of developing HF in the low-increasing trajectory group was found to be higher than in the low-stable trajectory group, highlighting the necessity of long-term management and control of aging status in clinical practice. In comparison, participants following high-decreasing trajectories exhibited a lower risk of HF compared to those on high-stable trajectories, whereas no significant differences were observed in the decreasing-increasing trajectory group. This suggested that individuals with initially high aging status may have reduced their HF risk by intervening to improve these levels. However, such improvements required sustained long-term efforts. Notably, the HF risk for participants in the high-decreasing trajectory remained higher than that for participants in the low-stable trajectory, indicating residual risk even after improvement. Furthermore, reducing aging status before significant accumulation of aging-related damage yielded less benefit in terms of HF incidence reduction compared to maintaining a low aging statusthroughout early life. Furthermore, we found that the association between baseline aging status and HF risk appeared more pronounced in younger participants compared to those aged 65 years and older at baseline. This may have been attributed to the fact that physiological functions (such as metabolic regulation, immune response, and organ reserve capacity) in individuals under 65 had not yet undergone irreversible decline, resulting in greater biological plasticity (28). This implied that aging-related characteristics (e.g., biological age percentile, aging status) in this age group had a more direct and sensitive impact on heart failure.

This study has several strengths. First, it was the first to investigate the association between BA and HF in a large-scale prospective cohort of Chinese adults aged 18 years and older. Second, BA was repeatedly measured, allowing us to identify six heterogeneous aging trajectories and to explore the longitudinal association between long-term changes in aging statusand subsequent HF risk. Third, we adjusted for a wide range of confounders—including smoking, alcohol consumption, and physical activity—thereby substantially reducing the potential for unmeasured residual confounding. Lastly, the traditional Cox model assumes “independent risks” and may overestimate the risk of the target event, especially in subgroups with high mortality. In this study, non-heart failure (HF) death was treated as a competing risk event, and a Fine-Gray competing risk model was constructed to calculate a more accurate hazard ratio (HR). Results of the competing risk analysis showed that compared with the primary results, there was no significant change in the associations between baseline aging status, biological age percentile trajectory patterns, and HF. Nonetheless, several limitations should be noted. First, all participants in our cohort are Chinese adults from the Kailuan community, with the majority being coal miners (30% are coal miners), which may limit the generalization of the conclusions to other populations. However, BA has demonstrated predictive utility for aging-related outcomes across multiple racial and ethnic groups, supporting the broader applicability of our findings. Second, the proportion of male participants in the cohort was relatively high. To mitigate sex-related bias, BA and aging status were calculated separately for men and women. Third, we were unable to classify HF into clinical subtypes. This study mainly recruited patients with heart failure with reduced ejection fraction (HFrEF), and the results may not be applicable to other types of heart failure. HF is a complex syndrome with heterogeneous pathophysiology, and future studies should explore whether specific BA patterns are differentially associated with HF subtypes. Lastly, in large cohort studies, perfect uniformity in follow-up intervals is difficult to achieve. As a result, minor misclassification of chronological age strata for a small number of participants may have occurred during BA percentile calculation. However, given the large sample size and the uniform distribution of such cases across age groups, the overall impact of this limitation is likely minimal.

## Conclusions

Using a DNNs model, we estimated BA to identify individual biological aging status and long-term aging trajectories. Compared with individuals exhibiting normal aging patterns, those with accelerated aging demonstrated an elevated risk of developing HF, while those with decelerated aging exhibited a reduced risk. Additionally, biological aging trajectories were significantly associated with subsequent HF risk. Specifically, individuals with consistently low aging status exhibited the lowest risk, whereas those transitioning from high to low aging status also experienced a reduction in risk. These findings emphasize the significant predictive value of BA in evaluating the risk of HF and reinforce the critical importance of promoting healthy aging within primary healthcare frameworks. Furthermore, these results establish a solid foundation for future experimental and clinical investigations aimed at elucidating the mechanisms and developing interventions to mitigate the impact of biological aging on HF progression.

## Data Availability

The raw data supporting the conclusions of this article will be made available by the authors, without undue reservation.
