# Supplementary material for: The association of biological age and its trajectory with incident heart failure: a cohort study from China
Source: Front Cardiovasc Med. 2026 Jan 22;12:1651743. doi: 10.3389/fcvm.2025.1651743 (PMC12872874; doi:10.3389/fcvm.2025.1651743)
Supplement: Supplementary file 2 [file Table2.docx]

**Supplementary Content**

**Supplementary Table 1.** Summary of methods, analytical wavelengths, and reaction types for clinical indicators

**Supplementary Table 2.** Basic Characteristics of 76,907 Participants According to the Baseline

**Supplementary Table 3.** Incremental Predictive Value of Biological Ageand Baseline Aging Status in Risk Assessment of Heart Failure in the 2006-2007 Survey

**Supplementary Table 4.** Association of Baseline Aging Status with the Risk of Heart Failure: Excluding Participants with Incident Heart Failure within the Initial One Years of Follow-up ^a^

**Supplementary Table 5.** Association of Baseline Aging Status with the Risk of Heart Failure treating Deaths as Competing Risk Events

**Supplementary Table 6.** Association of Biological Age Percentile Trajectory Patterns with the Risk of Heart Failure: Excluding Participants with Incident Heart Failure within the Initial One Years of Follow-up ^a^

**Supplementary Table 7.** Association of Biological Age Percentile Trajectory Patterns with the Risk of Heart Failure Treating Deaths as Competing Risk Events

**Supplementary Table 8.** Basic Characteristics of 32 indicators According to the Baseline

**Supplementary Table 9** Group-Based Trajectory Model Quality Metrics

**Supplementary Figure 1.** Stratified analysis for the association of Baseline Aging Status with the Risk of Heart Failure. Abbreviations: CI, confidence interval; HR, hazard ratio. Aging deceleration, less than the first biological age quartile; aging normal, ranged from the second to third biological age quartile; aging acceleration, higher than the third biological age quantile

**Supplementary Table 1. Summary of methods, analytical wavelengths, and reaction types for clinical indicators**

|  | Method | Analytical wavelength | Reaction type |
| --- | --- | --- | --- |
| TC, mmol/L | CHOD-PAP Method | Primary: 505nm Secondary: 700nm | End Point |
| TG, mmol/L | GPO-PAP Method | Primary: 500nm Secondary: 660nm | End Point |
| HDL-c, mmol/L | Direct Method - Selective Inhibition Method | Primary: 600nm Secondary: 700nm | End Point |
| LDL-c, mmol/L | Direct Method - Surfactant Depletion Method | Primary: 600nm Secondary: 700nm | End Point |
| FBG, mmol/L | Hexokinase Method | Primary: 340nm Secondary: 405nm | End Point |
| Hs-CRP, mg/L | Ultra-sensitive Latex-enhanced Immuno Turbidimetric Method | Primary: 570nm Secondary: 800nm | End Point |
| TBIL, μmol/L | Oxidation Method | Primary: 450nm Secondary: 546nm | End Point |
| ALT, U/L | Alanine Substrate Method | Primary: 340nm Secondary: 405nm | Rate |
| SUA, μmol/L | Uricase Method | Primary: 546nm Secondary: 700nm | End Point |
| Urea, mmol/L | Urease-Glutamate Dehydrogenase Method | Primary: 340nm Secondary: 405nm | Rate |
| Cr, μmol/L | Creatine Kinase Method | Primary: 546nm Secondary: 700nm | End Point |
| NEUT, *10^9^/L | Flow Cytometry |  |  |
| RBC, *10^12^/L | Electrical Impedance Method |  |  |
| WBC, *10^9^/L | Flow Cytometry |  |  |
| LYM, *10^9^/L | Flow Cytometry |  |  |
| MON, *10^9^/L | Flow Cytometry |  |  |
| PLT, *10^9^/L | Electrical Impedance Method |  |  |
| HGB, g/L | SLS-Hb Method |  |  |
| HCT | Instrument Calculation Method |  |  |
| MCV, fL | Electrical Impedance Method |  |  |
| PDW, % | Instrument Calculation Method |  |  |
| MCH, pg | Instrument Calculation Method |  |  |
| MCHC, g/L | Instrument Calculation Method |  |  |
| MPV, fL | Electrical Impedance Method |  |  |
| PCT, | Instrument Calculation Method |  |  |
| GRA% | Flow Cytometry |  |  |
| MON% | Flow Cytometry |  |  |
| LYM% | Flow Cytometry |  |  |

TC, total cholesterol, TG triglyceride; HDL-c, high-density lipoprotein; LDL, low-density lipoprotein; FBG, fasting blood glucose; Hs-CRP, high-sensitivity C-reactive protein; TBIL, Total bilirubin; ALT, alanine aminotransferase; SUA, serum uric acid; Cr, creatinine; NEUT, neutrophil count; RBC, red blood cell count; WBC, white blood cell count; LYM, lymphocyte; MON, monocytes; PLT, platelet count; HGB, hemoglobin; HCT, hematocrit; MCV, mean corpuscular volume; PDW, platelet distribution width; MCH, mean corpuscular hemoglobin; MCHC, mean corpuscular hemoglobin concentration; MPV, mean platelet volume; PCT, platelet crit; GRA, granulocyte.

**Supplementary Table 2. Basic Characteristics of 76,908 Participants According to the Baseline Aging Status ^a^**

| **Basic Characteristic** | **Aging deceleration** | **Aging normal** | **Aging acceleration** | **P value** |
| --- | --- | --- | --- | --- |
| No. of participants (%) | 19,186 (24.95) | 38,508 (50.07) | 19,214 (24.98) |  |
| Chronological age, mean (SD), y | 51.2 ± 12.3 | 51.3 ± 12.3 | 51.3 ± 12.4 | 0.8675 |
| Biological Age, mean (SD), y | 43.8 ± 5.9 | 51.2 ± 6.4 | 58.8 ± 6.9 | <.0001 |
| Gender, No. (%) |  |  |  | 0.9517 |
| Female | 3,927 (20.5) | 7,924 (20.6) | 3,942 (20.5) |  |
| Male | 15,259 (79.5) | 30,584 (79.4) | 15,272 (79.5) |  |
| Educational level, No. (%) |  |  |  | <.0001 |
| High school or below | 17,523 (91.3) | 36,136 (93.8) | 18,200 (94.7) |  |
| College or above | 1,663 (8.7) | 2,372 (6.2) | 1,014 (5.3) |  |
| Occupation, No. (%) |  |  |  | 0.122 |
| Coal miner | 5,983 (31.2) | 12,154 (31.6) | 5,904 (30.7) |  |
| Other | 13,203 (68.8) | 26,354 (68.4) | 13,310 (69.3) |  |
| Physical activity, No. (%) |  |  |  | 0.0135 |
| Low intensity | 1,674 (8.7) | 3,249 (8.4) | 1,579 (8.2) |  |
| Moderate intensity | 14,606 (76.1) | 29,245 (75.9) | 14,495 (75.4) |  |
| High intensity | 2,906 (15.1) | 6,014 (15.6) | 3,140 (16.3) |  |
| Smoking status, No. (%) |  |  |  | 0.0148 |
| Never | 11,490 (59.9) | 23,392 (60.7) | 11,573 (60.2) |  |
| Quit | 1,119 (5.8) | 2,116 (5.5) | 1,177 (6.1) |  |
| Currently | 6,577 (34.3) | 13,000 (33.8) | 6,464 (33.6) |  |
| Alcohol consumption status, No. (%) |  |  |  | 0.4619 |
| Never | 11,463 (59.7) | 22,987 (59.7) | 11,348 (59.1) |  |
| Quit | 727 (3.8) | 1,467 (3.8) | 773 (4.0) |  |
| Currently | 6,996 (36.5) | 14,054 (36.5) | 7,093 (36.9) |  |
| Salt intake, No. (%) |  |  |  | 0.0048 |
| Low salt intake | 1,860 (9.7) | 3,377 (8.8) | 1,689 (8.8) |  |
| Moderate salt intake | 15,323 (79.9) | 31,072 (80.7) | 15,481 (80.6) |  |
| High salt intake | 2,003 (10.4) | 4,059 (10.5) | 2,044 (10.6) |  |
| Income level, No. (%) |  |  |  | <.0001 |
| <$132/month | 17,773 (92.6) | 36,186 (94.0) | 18,037 (93.9) |  |
| ≥$132/month | 1,413 (7.4) | 2,322 (6.0) | 1,177 (6.1) |  |

^a^ Aging deceleration, less than the first biological age quartile; aging normal, ranged from the second to third biological age quartile; aging acceleration, higher than the third biological age quantile.

**Supplementary Table 3. Incremental Predictive Value of Biological Ageand Baseline Aging Status in Risk Assessment of Heart Failure in the 2006-2007 Survey**

| **Model** | **C-Statistic (95%CI)** | **P value** | **NRI (95%CI)** | **P value** |
| --- | --- | --- | --- | --- |
| Basic model | 0.7411 (0.7319-0.7504) |  | Ref |  |
| Basic model + biological Age | 0.7489 (0.7398-0.7580) | <0.0001 | 0.1860 (0.1451-0.2270) | <0.0001 |
| Basic model + baseline aging status | 0.7427 (0.7335-0.7520) | <0.0001 | 0.0143 (-0.0269,0.0554) | 0.4973 |

Abbreviations: NRI, net reclassification improvement; CI, confidence interval

Basic model included chronological age, gender, education level (high school or below or college or above), occupation (coal miner or other), physical activity (low intensity, moderate intensity, or high intensity), smoking status (never, quit, or currently), alcohol consumption (never, quit, or currently), salt intake (low salt intake, moderate salt intake, or high salt intake), and income level (income <1000 Chinese Yuan [$132]/month or income ≥1000 Chinese Yuan/month)

**Supplementary Table 4. Association of Baseline Aging Status with the Risk of Heart Failure: Excluding Participants with Incident Heart Failure within the Initial One Years of Follow-up ^a^**

| **Characteristics** | **Baseline aging status, HR (95% CI)** | | |
| --- | --- | --- | --- |
|  | **Aging deceleration** | **Aging normal** | **Aging acceleration** |
| **Heart failure** |  |  |  |
| Cases, No. (%) | 422 (2.21) | 1118 (2.91) | 715 (3.73) |
| Incident rate, per 1000 person-years | 1.47 | 1.96 | 2.55 |
| Model 1 | 0.75 (0.67-0.83) | Ref | 1.30 (1.18-1.43) |
| Model 2 | 0.74 (0.66-0.83) | Ref | 1.30 (1.18-1.43) |

Abbreviations: CI, confidence interval; HR, hazard ratio.

^a^ Excluding participants with incident Heart Failure within the initial one years of follow-up (N=76,664).

^b^ Model 1 adjusted for chronological age and gender.

^c^ Model 2 included covariates in model 1 and education level (high school or below or college or above), occupation (coal miner or other), physical activity (low intensity, moderate intensity, or high intensity), smoking status (never, quit, or currently), alcohol consumption (never, quit, or currently), salt intake (low salt intake, moderate salt intake, or high salt intake), and income level (income <1000 Chinese Yuan [$132]/month or income ≥1000 Chinese Yuan/month).

**Supplementary Table 5. Association of Baseline Aging Status with the Risk of Heart Failure treating Deaths as Competing Risk Events**

| **Models** | **Baseline aging status, HR (95% CI)** | | |
| --- | --- | --- | --- |
|  | **Aging deceleration** | **Aging normal** | **Aging acceleration** |
| Model 1 ^a^ | 0.77 (0.69-0.86) | Ref | 1.28 (1.17-1.41) |
| Model 2 ^b^ | 0.77 (0.69-0.86) | Ref | 1.28 (1.17-1.40) |

Abbreviations: CI, confidence interval; HR, hazard ratio.

^a^ Model 1 adjusted for chronological age and gender.

^b^ Model 2 included covariates in model 1 and education level (high school or below or college or above), occupation (coal miner or other), physical activity (low intensity, moderate intensity, or high intensity), smoking status (never, quit, or currently), alcohol consumption (never, quit, or currently), salt intake (low salt intake, moderate salt intake, or high salt intake), and income level (income <1000 Chinese Yuan [$132]/month or income ≥1000 Chinese Yuan/month).

**Supplementary Table 6. Association of Biological Age Percentile Trajectory Patterns with the Risk of Heart Failure: Excluding Participants with Incident Heart Failure within the Initial One Years of Follow-up ^a^**

| **Characteristics** | **Aging trajectory patterns, HR (95% CI)** | | | | | |
| --- | --- | --- | --- | --- | --- | --- |
|  | **Low-stable** | **Increasing-decreasing** | **Low-increasing** | **High-decreasing** | **Decreasing-increasing** | **High-stable** |
| **Heart failure** |  |  |  |  |  |  |
| Cases, No. (%) | 148 (1.47) | 74 (1.63) | 114 (1.88) | 117 (1.95) | 63 (2.04) | 292 (2.54) |
| Incident rate, per 1000 person-years | 1.27 | 1.42 | 1.63 | 1.69 | 1.78 | 2.24 |
| Model 1 ^b^ | Ref | 1.13 (0.86-1.50) | 1.30 (1.02-1.66) | 1.36 (1.07-1.73) | 1.45 (1.08-1.95) | 1.78 (1.46-2.17) |
| Model 2 ^c^ | Ref | 1.13 (0.86-1.49) | 1.30 (1.02-1.66) | 1.35 (1.06-1.72) | 1.45 (1.08-1.94) | 1.77 (1.46-2.16) |

Abbreviations: CI, confidence interval; HR, hazard ratio.

^a^ Excluding participants with incident heart failure within the initial one years of follow-up (N=41,269).

^b^ Model 1 adjusted for chronological age and gender.

^c^ Model 2 included covariates in model 1 and education level (high school or below or college or above), occupation (coal miner or other), physical activity (low intensity, moderate intensity, or high intensity), smoking status (never, quit, or currently), alcohol consumption (never, quit, or currently), salt intake (low salt intake, moderate salt intake, or high salt intake), and income level (income <1000 Chinese Yuan [$132]/month or income ≥1000 Chinese Yuan/month).

**Supplementary Table 7. Association of Biological Age Percentile Trajectory Patterns with the Risk of Heart Failure Treating Deaths as Competing Risk Events**

| **Models** | **Aging trajectory patterns, HR (95% CI)** | | | | | |
| --- | --- | --- | --- | --- | --- | --- |
|  | **Low-stable** | **Increasing-decreasing** | **Low-increasing** | **High-decreasing** | **Decreasing-increasing** | **High-stable** |
| Model 1 ^a^ | Ref | 1.11 (0.85-1.46) | 1.28 (1.01-1.62) | 1.30 (1.03-1.65) | 1.41 (1.06-1.88) | 1.75 (1.44-2.12) |
| Model 2 ^b^ | Ref | 1.11 (0.85-1.46) | 1.28 (1.01-1.63) | 1.29 (1.02-1.64) | 1.40 (1.05-1.87) | 1.74 (1.44-2.11) |

Abbreviations: CI, confidence interval; HR, hazard ratio.

^a^ Model 1 adjusted for chronological age and gender.

^b^ Model 2 included covariates in model 1 and education level (high school or below or college or above), occupation (coal miner or other), physical activity (low intensity, moderate intensity, or high intensity), smoking status (never, quit, or currently), alcohol consumption (never, quit, or currently), salt intake (low salt intake, moderate salt intake, or high salt intake), and income level (income <1000 Chinese Yuan [$132]/month or income ≥1000 Chinese Yuan/month).

**Supplementary Table 8. Basic Characteristics of 32 indicators According to the Baseline**

| **Basic Characteristic** | **Aging deceleration** | **Aging normal** | **Aging acceleration** | **P value** |
| --- | --- | --- | --- | --- |
| No. of participants (%) | 19,186 (24.95) | 38,508 (50.07) | 19,214 (24.98) |  |
| SBP (mmHg, mean [SD]) | 120.53 (16.15) | 131.25 (19.64) | 140.32 (21.18) | <0.0001 |
| DBP (mmHg, mean [SD]) | 80.08 (10.89) | 84.78 (11.66) | 84.76 (11.18) | <0.0001 |
| BMI (kg/m^2^, mean [SD]) | 24.82 (3.70) | 25.18 (3.32) | 25.02 (3.44) | <0.0001 |
| WHR (mean [SD]) | 0.88 (0.07) | 0.90 (0.07) | 0.90 (0.07) | <0.0001 |
| FBG (mmol/L, mean [SD]) | 5.14 (1.03) | 5.52 (1.66) | 5.64 (2.02) | <0.0001 |
| TC (mmol/L, mean [SD]) | 4.70 (1.06) | 5.02 (1.13) | 5.01 (1.19) | <0.0001 |
| TG (mmol/L, median [IQR]) | 1.65 (1.50) | 1.72 (1.39) | 1.62 (1.17) | <0.0001 |
| HDL-C (mmol/L, mean [SD]) | 1.48 (0.35) | 1.56 (0.39) | 1.63 (0.45) | <0.0001 |
| LDL-C (mmol/L, mean [SD]) | 2.37 (0.74) | 2.39 (0.88) | 2.35 (1.02) | <0.0001 |
| ALT (U/L, mean [SD]) | 22.98 (19.20) | 21.15 (14.78) | 19.18 (12.43) | <0.0001 |
| TBIL (μmol/L, mean [SD]) | 12.68 (5.47) | 13.14 (5.53) | 13.66 (5.93) | <0.0001 |
| Cr (μmol/L, mean [SD]) | 92.85 (27.61) | 90.81 (23.75) | 92.72 (25.10) | <0.0001 |
| BUN (mmol/L, mean [SD]) | 5.78 (1.51) | 5.46 (1.53) | 5.74 (1.55) | <0.0001 |
| PDW (%, mean [SD]) | 13.79 (1.69) | 13.53 (1.81) | 13.58 (2.05) | <0.0001 |
| UA (μmol/L, mean [SD]) | 285.00 (80.24) | 282.75 (80.34) | 298.30 (86.30) | <0.0001 |
| hs-CRP (mg/L, median [IQR]) | 0.60 (0.23,1.40) | 0.74 (0.30,1.90) | 1.13 (0.40,3.26) | <0.0001 |
| WBC (×10^9^ /L, mean [SD]) | 6.71 (1.63) | 6.56 (1.60) | 6.43 (1.55) | <0.0001 |
| NEUT (×10^9^ /L, mean [SD]) | 3.92 (1.21) | 3.91 (1.20) | 3.87 (1.16) | 0.0045 |
| MPV (fL, mean [SD]) | 7.59 (0.79) | 7.53 (0.82) | 7.57 (0.88) | <0.0001 |
| PCT (median [IQR]) | 0.16 (0.13,0.19) | 0.15 (0.12,0.18) | 0.14 (0.11,0.17) | <0.0001 |
| GRA% (mean [SD]) | 56.55 (7.71) | 57.77 (7.89) | 58.51 (8.06) | <0.0001 |
| MON (×10^9^ /L, median [IQR]) | 0.40 (0.30,0.50) | 0.40 (0.30,0.50) | 0.30 (0.20,0.50) | <0.0001 |
| PLT (×10^9^ /L, mean [SD]) | 214.85 (53.03) | 205.94 (54.78) | 193.62 (54.32) | <0.0001 |
| HGB (g/L, mean [SD]) | 152.02 (15.63) | 150.08 (15.31) | 146.95 (14.93) | <0.0001 |
| RBC (×10^12^/L, mean [SD]) | 5.05 (0.51) | 4.92 (0.49) | 4.82 (0.49) | <0.0001 |
| MON% (mean [SD]) | 6.74 (2.22) | 6.65 (2.27) | 6.46 (2.26) | <0.0001 |
| MCV (fL, mean [SD]) | 89.18 (6.33) | 89.59 (6.56) | 89.77 (6.50) | <0.0001 |
| MCHC (g/L, mean [SD]) | 339.32 (22.72) | 341.53 (22.50) | 341.33 (21.68) | <0.0001 |
| MCH (pg, mean [SD]) | 30.19 (2.06) | 30.54 (2.20) | 30.59 (2.25) | <0.0001 |
| HCT (mean [SD]) | 0.45 (0.05) | 0.44 (0.05) | 0.43 (0.05) | <0.0001 |
| LYM (×10^9^ /L, mean [SD]) | 2.38 (0.64) | 2.26 (0.64) | 2.18 (0.67) | <0.0001 |
| LYM% (mean [SD]) | 36.70 (7.16) | 35.56 (7.34) | 35.02 (7.66) | <0.0001 |

Abbreviations: ALT, alanine aminotransferase; BMI, body mass index; Cr, creatinine; DBP, diastolic blood pressure; FBG, fasting blood glucose; HCT, hematocrit; HDL-C, high density lipoprotein cholesterol; HGB, hemoglobin; hs-CRP, hypersensitive C-reactive protein; IQR, interquartile range; LDL-C, low density lipoprotein cholesterol; LYM, lymphocyte; LYM%, the percentage of lymphocyte; MCH, mean corpuscular hemoglobin; MCHC, mean corpuscular hemoglobin concentration; MCV, mean corpuscular volume; MON, monocytes; MON%, the percentage of monocytes; MPV, mean platelet volume; NEUT, neutrophil count; NEUT%, the percentage of neutrophil; PCT, platelet crit; PDW, platelet distribution width; PLT, platelet count; RBC, red blood cell count; SBP, systolic blood pressure; SD, standard deviation; TBIL, Total bilirubin; TC, total cholesterol, TG triglyceride; UA, uric acid; WBC, white blood cell count; WHR, waist-to-hip ratio.

**Supplementary Table 9. Group-Based Trajectory Model Quality Metrics**

| **Function order for each group** | | | | | | **Meaningful** | **BIC** | **AvePP** | **MPpG (%)** |
| --- | --- | --- | --- | --- | --- | --- | --- | --- | --- |
| **2 Trajectory** | | | | | |  |  |  |  |
| 1 | 2 |  |  |  |  | N |  |  |  |
| 2 | 1 |  |  |  |  | N |  |  |  |
| **3 Trajectory** | | | | | |  |  |  |  |
| 1 | 1 | 1 |  |  |  | Y | 14377.19 | 0.798 | 12.10 |
| 1 | 2 | 1 |  |  |  | Y | 14329.33 | 0.813 | 10.20 |
| 1 | 2 | 2 |  |  |  | Y | 14313.09 | 0.811 | 10.80 |
| 2 | 1 | 1 |  |  |  | Y | 14375.26 | 0.798 | 12.20 |
| 2 | 2 | 1 |  |  |  | Y | 14258.81 | 0.812 | 11.30 |
| 2 | 2 | 2 |  |  |  | Y | 14243.61 | 0.807 | 12.50 |
| 1 | 1 | 2 |  |  |  | N |  |  |  |
| 2 | 1 | 2 |  |  |  | N |  |  |  |
| **4 Trajectory** | | | | | |  |  |  |  |
| 1 | 1 | 2 | 2 |  |  | Y | 13559.98 | 0.742 | 18.70 |
| 1 | 2 | 2 | 1 |  |  | Y | 13292.99 | 0.749 | 20.10 |
| 2 | 1 | 1 | 2 |  |  | Y | 13616.01 | 0.733 | 20.00 |
| 2 | 1 | 2 | 2 |  |  | Y | 13560.98 | 0.744 | 18.20 |
| 2 | 2 | 1 | 2 |  |  | Y | 13420.11 | 0.755 | 16.90 |
| 2 | 2 | 2 | 1 |  |  | Y | 13293.60 | 0.749 | 20.10 |
| 1 | 1 | 1 | 1 |  |  | N |  |  |  |
| 1 | 1 | 1 | 2 |  |  | N |  |  |  |
| 1 | 1 | 2 | 1 |  |  | N |  |  |  |
| 1 | 2 | 1 | 1 |  |  | N |  |  |  |
| 1 | 2 | 1 | 2 |  |  | N |  |  |  |
| 1 | 2 | 2 | 2 |  |  | N |  |  |  |
| 2 | 1 | 1 | 1 |  |  | N |  |  |  |
| 2 | 1 | 2 | 1 |  |  | N |  |  |  |
| 2 | 2 | 1 | 1 |  |  | N |  |  |  |
| 2 | 2 | 2 | 2 |  |  | N |  |  |  |
| **5 Trajectory** | | | | | |  |  |  |  |
| 1 | 2 | 1 | 2 | 2 |  | Y | 12474.22 | 0.751 | 9.00 |
| 2 | 1 | 1 | 1 | 2 |  | Y | 12637.26 | 0.747 | 7.00 |
| 2 | 2 | 1 | 2 | 1 |  | Y | 12362.78 | 0.756 | 8.70 |
| 1 | 1 | 1 | 1 | 1 |  | N |  |  |  |
| 1 | 1 | 1 | 1 | 2 |  | N |  |  |  |
| 1 | 1 | 1 | 2 | 1 |  | N |  |  |  |
| 1 | 1 | 1 | 2 | 2 |  | N |  |  |  |
| 1 | 1 | 2 | 1 | 1 |  | N |  |  |  |
| 1 | 1 | 2 | 1 | 2 |  | N |  |  |  |
| 1 | 1 | 2 | 2 | 1 |  | N |  |  |  |
| 1 | 1 | 2 | 2 | 2 |  | N |  |  |  |
| 1 | 2 | 1 | 1 | 1 |  | N |  |  |  |
| 1 | 2 | 1 | 1 | 2 |  | N |  |  |  |
| 1 | 2 | 1 | 2 | 1 |  | N |  |  |  |
| 1 | 2 | 2 | 1 | 1 |  | N |  |  |  |
| 1 | 2 | 2 | 1 | 2 |  | N |  |  |  |
| 1 | 2 | 2 | 2 | 1 |  | N |  |  |  |
| 1 | 2 | 2 | 2 | 2 |  | N |  |  |  |
| 2 | 1 | 1 | 1 | 1 |  | N |  |  |  |
| 2 | 1 | 1 | 2 | 1 |  | N |  |  |  |
| 2 | 1 | 1 | 2 | 2 |  | N |  |  |  |
| 2 | 1 | 2 | 1 | 1 |  | N |  |  |  |
| 2 | 1 | 2 | 1 | 2 |  | N |  |  |  |
| 2 | 1 | 2 | 2 | 1 |  | N |  |  |  |
| 2 | 1 | 2 | 2 | 2 |  | N |  |  |  |
| 2 | 2 | 1 | 1 | 1 |  | N |  |  |  |
| 2 | 2 | 1 | 1 | 2 |  | N |  |  |  |
| 2 | 2 | 1 | 2 | 2 |  | N |  |  |  |
| 2 | 2 | 2 | 1 | 1 |  | N |  |  |  |
| 2 | 2 | 2 | 1 | 2 |  | N |  |  |  |
| 2 | 2 | 2 | 2 | 1 |  | N |  |  |  |
| 2 | 2 | 2 | 2 | 2 |  | N |  |  |  |
| **6 Trajectory** | | | | | |  |  |  |  |
| 1 | 2 | 1 | 2 | 2 | 2 | Y | 9845.68 | 0.771 | 13.34 |
| 1 | 1 | 2 | 2 | 2 | 2 | Y | 9845.68 | 0.771 | 13.35 |
| 2 | 1 | 1 | 2 | 2 | 2 | Y | 9845.68 | 0.771 | 13.35 |
| 1 | 2 | 2 | 2 | 1 | 2 | Y | 9882.21 | 0.772 | 9.64 |
| 1 | 1 | 2 | 2 | 1 | 2 | Y | 9888.37 | 0.772 | 9.62 |
| 1 | 2 | 1 | 1 | 2 | 2 | Y | 9888.37 | 0.772 | 9.62 |
| 1 | 2 | 2 | 1 | 2 | 2 | Y | 9944.77 | 0.770 | 9.98 |
| 1 | 2 | 2 | 2 | 2 | 1 | Y | 9944.77 | 0.770 | 9.98 |
| 2 | 1 | 2 | 1 | 2 | 2 | Y | 9944.77 | 0.770 | 9.98 |
| 2 | 2 | 2 | 1 | 2 | 1 | Y | 10705.57 | 0.736 | 10.53 |
| 1 | 1 | 1 | 1 | 1 | 1 | N |  |  |  |
| 1 | 1 | 1 | 1 | 1 | 2 | N |  |  |  |
| 1 | 1 | 1 | 1 | 2 | 1 | N |  |  |  |
| 1 | 1 | 1 | 1 | 2 | 2 | N |  |  |  |
| 1 | 1 | 1 | 2 | 1 | 1 | N |  |  |  |
| 1 | 1 | 1 | 2 | 1 | 2 | N |  |  |  |
| 1 | 1 | 1 | 2 | 2 | 1 | N |  |  |  |
| 1 | 1 | 1 | 2 | 2 | 2 | N |  |  |  |
| 1 | 1 | 2 | 1 | 1 | 1 | N |  |  |  |
| 1 | 1 | 2 | 1 | 1 | 2 | N |  |  |  |
| 1 | 1 | 2 | 1 | 2 | 1 | N |  |  |  |
| 1 | 1 | 2 | 1 | 2 | 2 | N |  |  |  |
| 1 | 1 | 2 | 2 | 1 | 1 | N |  |  |  |
| 1 | 1 | 2 | 2 | 2 | 1 | N |  |  |  |
| 1 | 2 | 1 | 1 | 1 | 1 | N |  |  |  |
| 1 | 2 | 1 | 1 | 1 | 2 | N |  |  |  |
| 1 | 2 | 1 | 1 | 2 | 1 | N |  |  |  |
| 1 | 2 | 1 | 2 | 1 | 1 | N |  |  |  |
| 1 | 2 | 1 | 2 | 1 | 2 | N |  |  |  |
| 1 | 2 | 1 | 2 | 2 | 1 | N |  |  |  |
| 1 | 2 | 2 | 1 | 1 | 1 | N |  |  |  |
| 1 | 2 | 2 | 1 | 1 | 2 | N |  |  |  |
| 1 | 2 | 2 | 1 | 2 | 1 | N |  |  |  |
| 1 | 2 | 2 | 2 | 1 | 1 | N |  |  |  |
| 1 | 2 | 2 | 2 | 2 | 2 | N |  |  |  |
| 2 | 1 | 1 | 1 | 1 | 1 | N |  |  |  |
| 2 | 1 | 1 | 1 | 1 | 2 | N |  |  |  |
| 2 | 1 | 1 | 1 | 2 | 1 | N |  |  |  |
| 2 | 1 | 1 | 1 | 2 | 2 | N |  |  |  |
| 2 | 1 | 1 | 2 | 1 | 1 | N |  |  |  |
| 2 | 1 | 1 | 2 | 1 | 2 | N |  |  |  |
| 2 | 1 | 1 | 2 | 2 | 1 | N |  |  |  |
| 2 | 1 | 2 | 1 | 1 | 1 | N |  |  |  |
| 2 | 1 | 2 | 1 | 1 | 2 | N |  |  |  |
| 2 | 1 | 2 | 1 | 2 | 1 | N |  |  |  |
| 2 | 1 | 2 | 2 | 1 | 1 | N |  |  |  |
| 2 | 1 | 2 | 2 | 1 | 2 | N |  |  |  |
| 2 | 1 | 2 | 2 | 2 | 1 | N |  |  |  |
| 2 | 1 | 2 | 2 | 2 | 2 | N |  |  |  |
| 2 | 2 | 1 | 1 | 1 | 1 | N |  |  |  |
| 2 | 2 | 1 | 1 | 1 | 2 | N |  |  |  |
| 2 | 2 | 1 | 1 | 2 | 1 | N |  |  |  |
| 2 | 2 | 1 | 1 | 2 | 2 | N |  |  |  |
| 2 | 2 | 1 | 2 | 1 | 1 | N |  |  |  |
| 2 | 2 | 1 | 2 | 1 | 2 | N |  |  |  |
| 2 | 2 | 1 | 2 | 2 | 1 | N |  |  |  |
| 2 | 2 | 1 | 2 | 2 | 2 | N |  |  |  |
| 2 | 2 | 2 | 1 | 1 | 1 | N |  |  |  |
| 2 | 2 | 2 | 1 | 1 | 2 | N |  |  |  |
| 2 | 2 | 2 | 1 | 2 | 2 | N |  |  |  |
| 2 | 2 | 2 | 2 | 1 | 1 | N |  |  |  |
| 2 | 2 | 2 | 2 | 1 | 2 | N |  |  |  |
| 2 | 2 | 2 | 2 | 2 | 1 | N |  |  |  |
| 2 | 2 | 2 | 2 | 2 | 2 | N |  |  |  |

Abbreviations: BIC, Bayesian Information Criterion; Avepp, Average Posterior Probability; MCRpG, Minimum Proportion per Group.


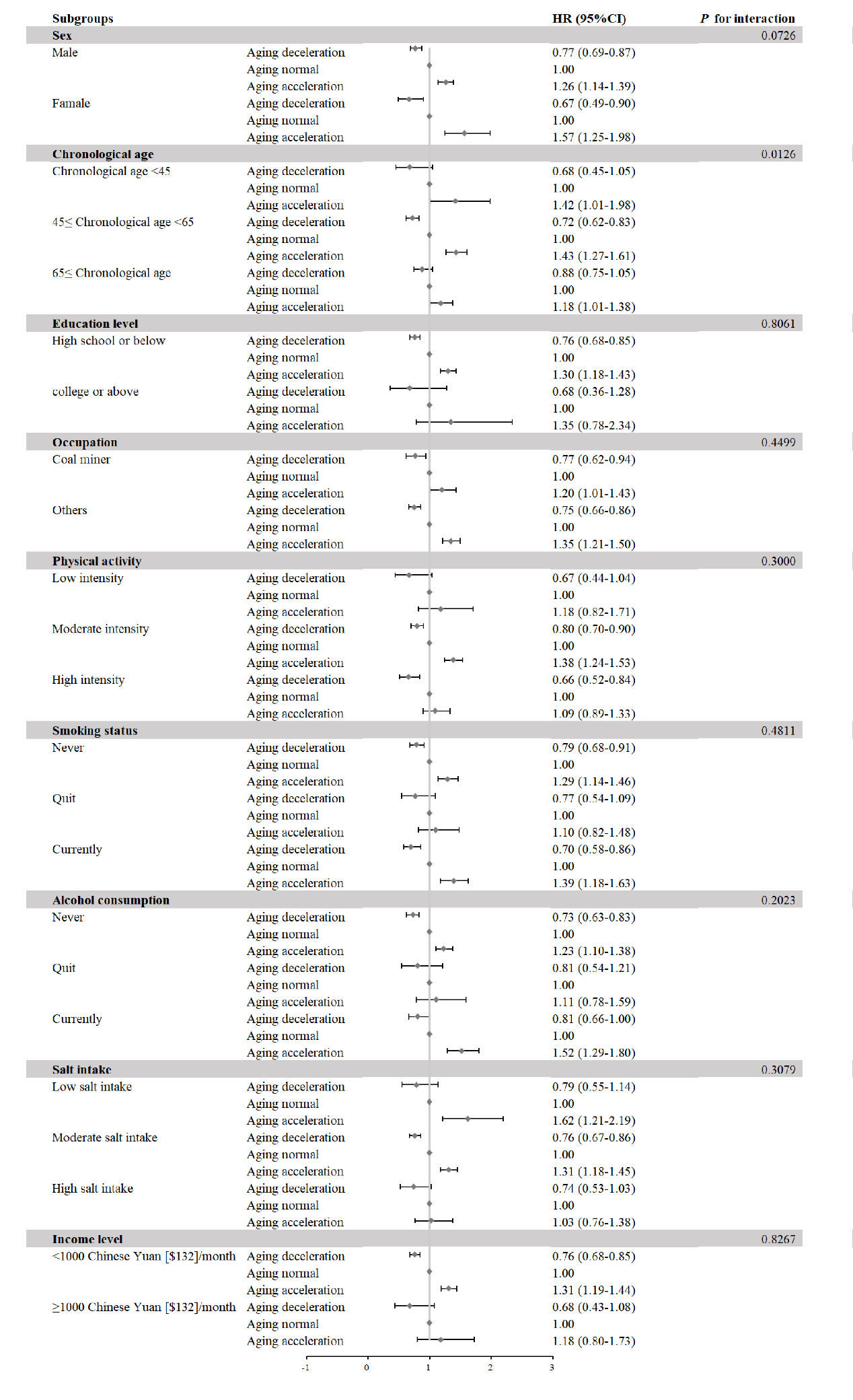


**Supplementary Figure 1.** Stratified analysis for the association of Baseline Aging Status with the Risk of Heart Failure. Abbreviations: CI, confidence interval; HR, hazard ratio. Aging deceleration, less than the first biological age quartile; aging normal, ranged from the second to third biological age quartile; aging acceleration, higher than the third biological age quantile
